# Association of hepatitis B virus DNA levels with efficacy and safety and the impact of antiviral therapy on prognosis in liver cancer patients receiving immune checkpoint inhibitors therapy: a systematic review and meta-analysis

**DOI:** 10.3389/fmicb.2025.1501139

**Published:** 2025-01-22

**Authors:** Hongxia Cui, Su Li, Wu Lv, Jing Xiang

**Affiliations:** ^1^Department of Pharmacy, Cancer Hospital of China Medical University, Liaoning Cancer Hospital and Institute, Shenyang, China; ^2^Department of General Surgery, Cancer Hospital of China Medical University, Liaoning Cancer Hospital and Institute, Shenyang, China

**Keywords:** liver cancer, hepatitis B virus DNA, immune checkpoint inhibitors, meta-analysis, antiviral therapy

## Abstract

**Background:**

The current evidence regarding the relationship between baseline hepatitis B virus (HBV) DNA levels and survival outcomes in liver cancer patients receiving immune checkpoint inhibitors (ICIs) remains inconsistent. Therefore, this review was intended to explore the impact of the baseline HBV-DNA level on the efficacy and safety of ICIs in patients with liver cancer.

**Methods:**

Relevant studies were identified through a comprehensive search in PubMed, EMBASE, Cochrane Library, and Web of Science up to August 1, 2024. The outcomes were hazard ratios (HRs) for overall survival (OS) and progression-free survival (PFS), as well as odds ratios (ORs) for objective response rate (ORR), disease control rate (DCR) and HBV reactivation (HBVr). Subgroup analysis, publication bias, and sensitivity analysis were conducted with STATA 14.0.

**Results:**

This meta-analysis comprised 17 articles involving a total of 2,130 patients. The pooled results demonstrated that high HBV DNA was associated with a worse OS (HR = 1.48 95% CI 1.11–1.96). Further subgroup analysis showed that there was no difference in OS between the high HBV DNA group and low HBV DNA group when all patients received antiviral treatment. No associations between baseline HBV DNA and PFS (HR = 1.08, 95% CI 0.90–1.29), ORR (OR = 0.91, 95% CI 0.65–1.28), or DCR (OR = 0.83, 95% CI 0.58–1.20) were observed. The risk of HBVr in the high HBV DNA group was lower than that in the low HBV DNA group (OR = 0.30, 95% CI 0.15–0.58), especially among patients who received antiviral therapy (OR = 0.42, 95% CI 0.18–0.98).

**Conclusion:**

High HBV DNA was associated with worse OS, but not with PFS, ORR, or DCR in liver cancer patients receiving ICIs. When patients were simultaneously treated with antiviral treatment, elevated HBV DNA level had no unfavorable impact on the efficacy of ICIs. Furthermore, the risk of HBVr in the high HBV-DNA group was lower than that in the low HBV DNA group. More prospective studies with larger sample sizes are essential to confirm the results.

## 1 Introduction

Primary liver cancer, subclassified into combined hepatocellular-cholangiocarcinoma (cHCC-ICC), intrahepatic cholangiocarcinoma (ICC), and hepatocellular carcinoma (HCC) (Hu et al., 2024), ranks as the sixth most common cancer worldwide (Sung et al., 2021). Specific agents, such as viral infections induced by hepatitis B virus (HBV) and hepatitis C virus (HCV), serve as crucial risk factors for the progression of liver cancer (Clements et al., 2020; Singal et al., 2020). The etiology of liver cancer differs by region. For Western patients, the primary contributors to liver cancer are HCV infection and alcohol misuse, whereas in China, chronic HBV infection is the main etiology, with nearly 85% of liver cancer patients having a history of HBV or currently being infected with HBV (Wang et al., 2017; Villanueva, 2019).

Immune checkpoint inhibitors (ICIs) therapy have revolutionized the treatment strategies for HCC with the rapid development of molecular biology and immunology (Sangro and Sarobe, 2021). In the CheckMate-040 study, all advanced HCC patients, including fifty-one patients with HBV, had similar tumor responses when they were treated with nivolumab (El-Khoueiry et al., 2017). The phase III IMbrave150 trial demonstrated that the efficacy and safety of the combination of atezolizumab and bevacizumab were superior to those of sorafenib in HCC (Finn et al., 2020). However, in the above trials, patients with high baseline HBV DNA level (>100 IU/mL or > 500 IU/mL) were excluded. The reason for the exclusion of patients with high HBV-DNA was that PD-1 inhibitors may trigger HBV reactivation (HBVr) (Cho et al., 2017; Lake, 2017). The occurrence of HBVr might negatively influence the survival of HCC patients by deteriorating liver function or impeding the continuation of potentially life-saving HCC treatment (Lee et al., 2021; Wu et al., 2012; Papatheodoridi et al., 2022). The impact of baseline HBV DNA levels on clinical outcomes and whether ICIs induce HBVr in HCC patients receiving anti-PD-1 therapy have not been assessed in most clinical trials. In clinical practice, a significant portion of patients with HBV-related cancer exhibited high HBV DNA at the time of diagnosis (Shi et al., 2013). Therefore, the impact of high HBV-DNA on the efficacy and safety of immunotherapy in liver cancer patients needs to be fully demonstrated.

Recently, several studies have investigated the efficacy and safety of using ICIs in liver cancer patients with high HBV DNA levels, but the results were inconsistent (An et al., 2022; Chen et al., 2020; Chen et al., 2023; Chen et al., 2022; Han et al., 2024; He et al., 2021; Hu et al., 2022; Lee et al., 2020; Liang et al., 2024; Pan et al., 2024; Pan et al., 2022; Shen et al., 2024; Sun et al., 2020; Wang et al., 2021; Wang et al., 2024; Yang et al., 2024; Yuan et al., 2021). For instance, several studies demonstrated a significant association between elevated HBV level and poor clinical outcomes (Chen et al., 2022; Liang et al., 2024), while other investigations reported no substantial correlation between HBV viral load and prognosis in liver cancer patients who received ICIs treatment (An et al., 2022; Chen et al., 2020; Chen et al., 2023; Pan et al., 2024; Pan et al., 2022; Shen et al., 2024; Sun et al., 2020). To the best of our knowledge, there has been no meta-analysis to compare the effectiveness and safety of high HBV DNA group and low HBV DNA group. To this end, we performed this meta-analysis on the basis of the current research status to clarify the impact of baseline HBV DNA levels on the effectiveness and safety of ICIs in liver cancer patients.

## 2 Methods

The review was prepared adhering to the Preferred Reporting Items for Systematic Reviews and Meta-analyses (PRISMA) guidelines (Liberati et al., 2009). The protocol for this review was pre-registered on PROSPERO (CRD42024578829).

### 2.1 Search strategy

A comprehensive literature search of PubMed, EMBASE, Cochrane Library, and Web of Science was conducted. The latest search date was August 1, 2024. The search terms included “Neoplasms” [Mesh], “Immune Checkpoint Inhibitors” [Mesh], “Hepatitis B virus” [Mesh], and their entry terms. See Supplementary Table S1 for the detailed search strategies. First, titles and abstracts were screened for relevance, and then the full texts were screened for available studies. In addition, to obtain eligible reports, we further scanned the reference lists of the included articles.

### 2.2 Selection criteria

The eligibility criteria based on the Population-Intervention-Control-Outcome-Study (PICOS) framework were as follows: (1) population: the patients were diagnosed with liver cancer and treated with ICIs, and patients were classified into two groups according to baseline HBV DNA; (2) intervention: patients with high baseline HBV DNA; (3) control: patients with low baseline HBV DNA; (4) outcomes: studies needed to provide at least one of the outcomes of interest: HRs for overall survival (OS), HRs for progression-free survival (PFS), odds ratios (ORs) for objective response rate (ORR), ORs for disease control rate (DCR), ORs for incidence of hepatitis B virus reactivation (HBVr), comparing patients with high HBV DNA and patients with low HBV DNA. (5) study design: prospective or retrospective studies published in English. Duplicate, case report, letter, comment, animal study, review or meta-analysis were excluded. If studies with duplicate patients were given, the most recent study was selected.

### 2.3 Data extraction and quality evaluation

Two investigators separately extracted the following relevant data: first author, publication year, region, cancer type, sample size, number of male and female patients, follow-up, treatment, use of antiviral treatment, cut-off value of HBV DNA, clinical outcomes, source of HRs, HRs for OS and/or PFS, number of complete response (CR), partial response (PR) and stable disease (SD) (ORR = CR + PR, DCR = CR + PR + SD), and number of HBVr. All included studies were retrospective cohort studies, and we evaluated their quality through the NOS score. The NOS ranged from 0 to 9, and a study with NOS score of 7 or above was considered high-quality (Stang, 2010).

### 2.4 Data analysis

The associations of HBV DNA with OS and PFS were estimated by pooled HRs and 95% CIs. If the multivariate and univariate analysis were simultaneously reported in studies, we prioritized the results of the multivariate analysis. If no HRs and 95% CIs were available in studies, we extracted data from the survival curves based on the methods proposed by Tierney et al. (2007). Combined ORs with 95% CIs were utilized to assess the predictive significance of HBV DNA for ORR, DCR, and HBVr. We combined outcomes utilizing either a random-effects model or a fixed-effects model based on the homogeneity among studies. Heterogeneity was assessed through Cochran’s Q and I^2^ tests. A random-effects model was applied when *p* < 0.1 or I^2^ > 50%, indicating significant heterogeneity.

In addition to the overall analysis, subgroup analyses were performed to investigate the associations of treatment method, cut-off value, source of HRs and antiviral therapy with outcomes. To assess the stability of the combined results, sensitivity analysis was conducted by omitting one study at a time. Publication bias assessment was performed using funnel plots, Begg’s test, and Egger’s test. Statistical analyses were performed with Stata 14.0, and a *p*-value of less than 0.05 was considered statistically significant.

## 3 Results

### 3.1 Literature search

**Figure 1:**
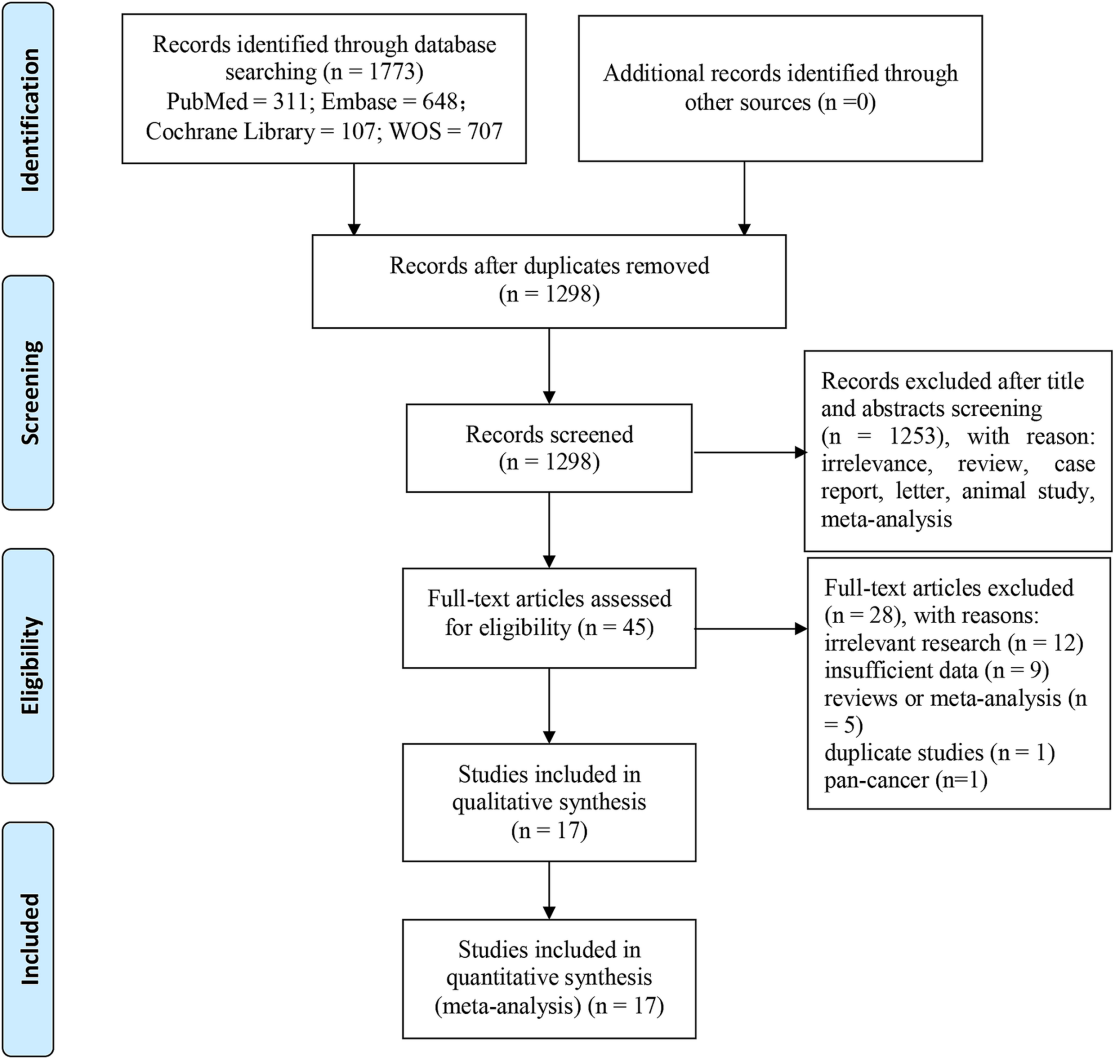
Procedure of literature screening.

A total of 1773 records were retrieved from PubMed (*n* = 311), Embase (*n* = 648), Web of Science (*n* = 707), and Cochrane Library (*n* = 107). After eliminating duplicates (*n* = 475), 1,298 articles were left. Following the review of titles and abstracts, we excluded 1,253 papers owing to irrelevance, animal studies, case reports, letters, comments, meta-analyses or reviews. The remaining 45 reports were further screened through full-text review. Among them, 28 studies were eliminated for the following reasons: irrelevant research (*n* = 12), no sufficient data (*n* = 9), reviews or meta-analysis (*n* = 5), overlapping patients (*n* = 1) and pan-cancer (*n* = 1). Ultimately, we recruited 17 articles involving 2,130 liver cancer patients. Figure 1 presents the process of literature screening.

### 3.2 Study characteristics and quality evaluation

All 17 studies were conducted in China, and were published between 2020 and 2024. The majority (81.2%) of the participants were males. Considering the category of liver cancer, all the studies focused on HCC, except for one study that included HCC and ICC. Regarding treatment approaches, 2 studies focused on anti-PD-1 monotherapy, 8 studies involved a combination of anti-PD-1 with other therapies, and the remaining 7 studies included either monotherapy or combination therapy. The cut-off value for HBV DNA varied. For instance, the cut-off was not specified in one study, it was 100 IU/mL in one study, 500 IU/mL in five studies, 1,000 IU/mL in one study, 2,000 IU/mL in six studies, 215 copy/ml in one study, and 1,000 copy/ml in one study. Among the studies included, 10 studies examined the effect of baseline HBV DNA on OS, whereas 8 focused on its impact on PFS in liver cancer patients receiving ICIs. The ORR, DCR and HBVr were reported in 6,6 and 8 studies, respectively. All included studies were retrospective cohort studies, and the NOS criteria was applied to evaluate quality. The NOS scores for the 10 studies ranged from 5 to 8, which suggested medium or high quality (Supplementary Table S2). Table 1 lists the characteristics of the 17 included studies.

**Table 1:** The main characteristics of the studies included in the meta-analysis.

Author	Year	Country	Cancer type	Sample size	Sex/man	Age (y)	Treatment	Cut-off	Outcomes	NOS
An et al. (2022)	2023	China	HCC	165	147	NA	Anti-PD-1 monotherapy/ combination therapy	500 IU/mL	PFS, OS, ORR, DCR	7
Chen et al. (2020)	2020	China	HCC	22	19	53 (36–71)^a^	Anti-PD-1 combination therapy	215 copy/mL	PFS, OS	8
Chen et al. (2023)	2023	China	HCC	149	128	NA	Anti-PD-1 combination therapy	500 IU/mL	PFS, OS, ORR, DCR, HBVr	8
Chen et al. (2022)	2022	China	HCC	49	NA	NA	Anti-PD-1 monotherapy	500 IU/mL	PFS, OS	5
Han et al. (2024)	2024	China	HCC	155	136	NA	Anti-PD-1/PD-L1monotherapy/combination therapy	NA	PFS	5
He et al. (2021)	2021	China	HCC	202	170	49 (19–74)^a^	Anti-PD-1 monotherapy/ combination therapy	500 IU/mL	HBVr	7
Hu et al. (2022)	2022	China	HCC	70	66	52.5 ± 12.2^b^	Anti-PD-1 combination therapy	2000 IU/mL	PFS, ORR, DCR, HBVr	5
Lee et al. (2020)	2020	China	HCC	60	50	NA	Anti-PD-1 monotherapy/ combination therapy	100 IU/mL	HBVr	6
Liang et al. (2024)	2024	China	HCC	44	38	NA	Anti-PD-1 monotherapy	1,000 copy/mL	RFS, OS	6
Pan et al. (2024)	2024	China	HCC	120	75	NA	Anti-PD-1 combination therapy	2000 IU/mL	PFS, OS, ORR, DCR, HBVr	5
Pan et al. (2022)	2022	China	HCC/ ICC	48	41	55.96 ± 9.72^b^	Anti-PD-1 combination therapy	NA	OS, ORR, DCR	5
Shen et al. (2024)	2023	China	HCC	119	109	57 (19-82)^a^	Anti-PD-1 combination therapy	2000 IU/mL	OS, HBVr	5
Sun et al. (2020)	2020	China	HCC	253	217	NA	Anti-PD-1 monotherapy/ combination therapy	2000 IU/mL	PFS, OS	6
Wang et al. (2021)	2021	China	HCC	157	140	NA	Anti-PD-1 monotherapy/ combination therapy	500 IU/mL	OS	7
Wang et al. (2024)	2024	China	HCC	218	194	53.35 ± 10.82^b^	Anti-PD-1 monotherapy/ combination therapy	1,000 IU/mL	HBVr	8
Yang et al. (2024)	2024	China	HCC	213	194	51.4 ± 10.4^b^	Anti-PD-1 combination therapy	2000 IU/mL	HBVr	7
Yuan et al. (2021)	2021	China	HCC	86	72	55^b^	Anti-PD-1 combination therapy	2000 IU/mL	ORR, DCR, HBVr	7

HCC, Hepatocellular carcinoma; ICC, Intrahepatic Cholangiocarcinoma; OS, overall survival; PFS, progression-free survival; ORR, objective response rate; DCR, disease control rate; HBVr, hepatitis B virus reactivation; UV, univariate analysis; ICIs, immune checkpoint inhibitors; NA, not available.

### 3.3 Baseline HBV DNA levels and OS

**Figure 2:**
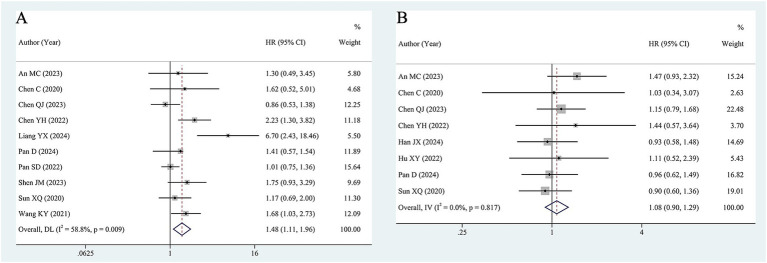
Forest plot of pooled HR for OS **(A)**, PFS **(B)**.

Ten studies, including 1,126 patients, assessed the significance of baseline HBV DNA levels in predicting the OS of liver cancer patients. Assessment of heterogeneity suggested significant heterogeneity (I^2^ = 58.8%, *p* = 0.009). Thus, a random-effects model was utilized to estimate the pooled effect. The pooled HR of 1.48 (95% CI 1.11–1.96) revealed that elevated HBV DNA was associated with worse OS in liver cancer patients who received ICIs (Figure 2A).

To identify potential sources of heterogeneity and factors that may affect the results, subgroup analyses were conducted based on the following factors: treatment method (monotherapy, combination therapy, and monotherapy or combination therapy), cut-off value (500 IU/m and ≥ 2000 IU/m), and antiviral treatment (all and partial). When patients were treated with monotherapy, high HBV DNA still significantly decreased OS (HR = 3.54, 95% CI 1.22–10.27), and there was a trend toward worse OS in the combination therapy group, this difference was not statistically significant (HR = 1.14, 95% CI 0.90–1.45). In terms of the cut-off value, in studies with cut-off value of 2000 IU/mL, patients with high HBV DNA had worse OS than those with low HBV DNA (HR = 1.39, 95% CI 1.02–1.91). However, in studies with cut-off value of 500 IU/mL, a significant difference was not detected (HR = 1.43, 95% CI 0.91–2.27). In cases where partial patients underwent antiviral treatment, high HBV DNA was related to poor prognosis (HR = 1.38, 95% CI 1.02–1.87). However, when all patients received antiviral therapy, high HBV DNA was not significantly associated with OS (HR = 1.51, 95% CI 0.85–2.68). Heterogeneity was not significantly reduced in the above subgroups. The results of the subgroup analyses are shown in Table 2 and Supplementary Figure S1.

**Table 2:** Subgroup analyses of baseline HBV DNA level for OS and PFS.

Subgroup	No. of studies	Sample size	Effects model	HR and 95%CI	Heterogeneity	
					I^2^ (%)	*p* value
OS	10	1,126	Random	1.48 (1.11–1.96)	58.8	0.009
Treatment method						
Combination therapy	5	458	Random	1.14 (0.90–1.45)	16.4	0.310
Monotherapy	2	93	Random	3.54 (1.22–10.27)	71.6	0.060
Mixed	3	575	Random	1.41 (1.01–1.97)	0	0.607
Cut-off criteria						
500	4	520	Random	1.43 (0.91–2.27)	60	0.058
2,000	3	492	Random	1.39 (1.02–1.91)	0	0.636
Antiviral treatment						
All	4	398	Random	1.51 (0.85–2.68)	81.3	0.001
Partial	4	548	Random	1.38 (1.02–1.87)	0	0.820
PFS	8	983	Fixed	1.08 (0.90–1.29)	0	0.817
Treatment method						
Combination therapy	4	361	Fixed	1.07 (0.83–1.39)	0	0.94
Monotherapy	1	49	Fixed	1.44 (0.57–3.64)	0	0
Mixed	3	573	Fixed	1.06 (0.82–1.37)	30.4	0.238
Cut-off criteria						
500	3	363	Fixed	1.29 (0.97–1.70)	0	0.701
2,000	3	443	Fixed	0.95 (0.72–1.25)	0	0.890
Antiviral treatment						
All	2	384	Random	1.14 (0.82–1.60)	0	0.934
Partial	3	373	Random	1.06 (0.83–1.37)	27.9	0.250

### 3.4 Baseline HBV DNA levels and PFS

As shown in Figure 2B, 8 studies with 983 patients investigated the impact of baseline HBV DNA on PFS. Cochran’s Q and I^2^ tests indicated no significant heterogeneity (I^2^ = 0%, *p* = 0.817). Consequently, a fixed-effects model was adopted. The pooled HR was 1.08 (95% CI 0.90–1.29), which indicated that there was no significant difference in PFS between patients with high HBV DNA and those with low HBV DNA.

To determine whether the association of HBV DNA with PFS would vary in accordance with certain factors, subgroup analysis was conducted. As shown in Table 2 and Supplementary Figure S2, the subgroup analyses also indicated that HBV DNA level was not associated with PFS regardless of the treatment method, cut-off value of HBV DNA or antiviral treatment. Additionally, there was no significant heterogeneity among studies in all subgroups.

### 3.5 Baseline HBV DNA levels and immunotherapy responses

**Figure 3:**
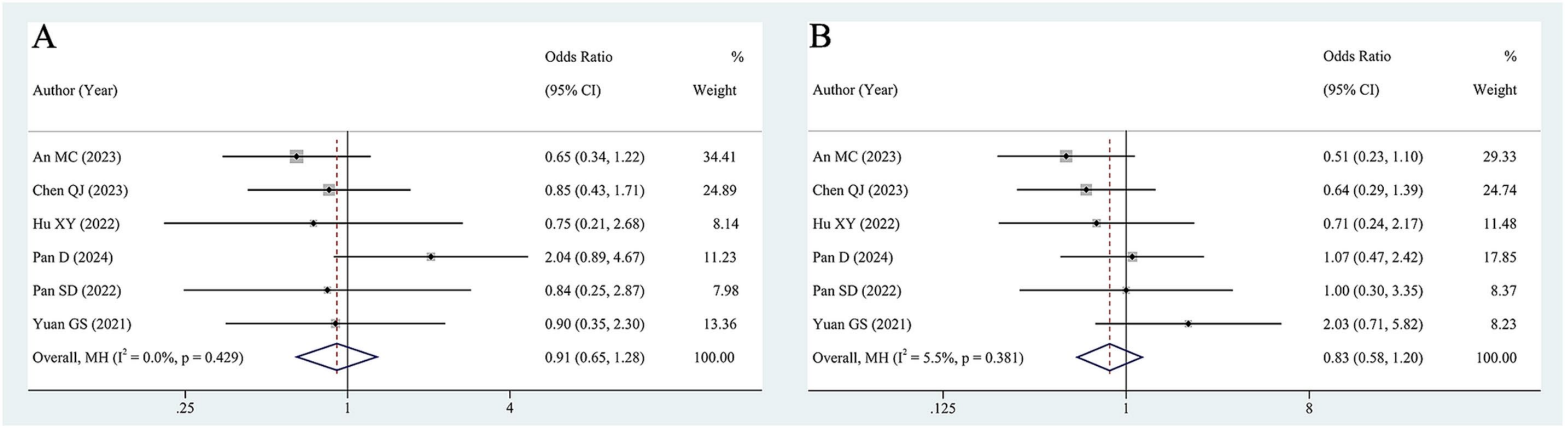
Forest plot of pooled OR for ORR **(A)**, DCR **(B)**.

Six studies (638 patients) examined the association between baseline HBV DNA and ORR. The pooled OR was 0.91 (95% CI 0.65–1.28, Figure 3A) according to a fixed-effects model (I^2^ = 0, *p* = 0.429), suggesting that baseline HBV DNA levels did not correlate significantly with the ORR. Furthermore, we pooled the data from 6 studies encompassing 638 patients to analyze the association between baseline HBV DNA and DCR. The degree of between-study heterogeneity was low (I^2^ = 5.5%, *p* = 0.381%), so the analysis was conducted using a fixed-effects model. The results revealed that there was no significant relationship between baseline HBV DNA and DCR (OR = 0.83, 95% CI 0.58–1.20, Figure 3B).

Considering that the treatment method, the cut-off value of HBV DNA and antiviral treatment might have an impact on the merged results, subgroup analyses for ORR and DCR were implemented. The pooled results of subgroup analyses suggested that patients with high baseline HBV DNA exhibited comparable ORR and DCR to those with low baseline HBV DNA in all subgroups, except for the subgroup with cut-off of 500 IU/mL, where the DCR of the high baseline HBV DNA group was lower than that of the low baseline HBV DNA group (OR = 0.57, 95% CI 0.33–0.98). The results of the subgroup analyses are shown in Table 3 and Supplementary Figures S3, S4.

**Table 3:** Subgroup analyses of baseline HBV DNA level for ORR and DCR.

Subgroup	No. of studies	Sample size	Effects model	OR and 95%CI	Heterogeneity	
					I^2^ (%)	*P* value
ORR	6	638	Fixed	0.91 (0.65–1.28)	0	0.429
Treatment method						
Combination therapy	5	473	Fixed	1.05 (0.70–1.58)	0	0.510
Mixed	1	165	Fixed	0.65 (0.34–1.22)	0	0
Cut-off criteria						
500	2	314	Fixed	0.73 (0.46–1.17)	0	0.562
2,000	3	276	Fixed	1.25 (0.73–2.17)	17.5	0.297
Antiviral treatment						
All	4	285	Random	0.85 (0.53–1.36)	0	0.997
Partial	2	353	Random	1.11 (0.36–3.42)	78.5	0.031
DCR	6	638	Fixed	0.83 (0.58–1.20)	5.5	0.381
Treatment method						
Combination therapy	5	473	Fixed	0.96 (0.63–1.47)	0	0.503
Mixed	1	165	Fixed	0.51 (0.23–1.10)	0	0
Cut-off criteria						
500	2	314	Fixed	0.57 (0.33–0.98)	0	0.685
2,000	3	276	Fixed	1.17 (0.68–2.03)	0	0.397
Antiviral treatment						
All	4	285	Fixed	0.93 (0.57–1.51)	7.1	0.358
Partial	2	353	Fixed	0.72 (0.41–1.25)	41.3	0.192

### 3.6 Baseline HBV DNA levels and HBVr

**Figure 4:**
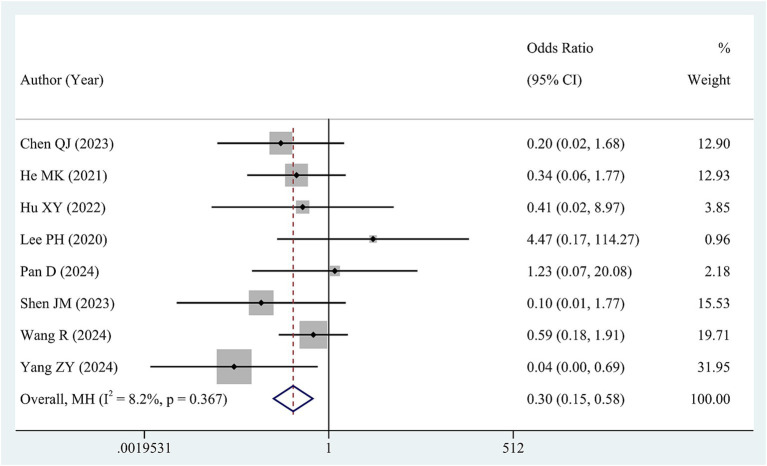
Forest plot of pooled OR for HBVr.

Data regarding HBVr was available from 8 studies involving a total of 1,237 patients. Low heterogeneity was observed in these studies (I^2^ = 8.2, *p* = 0.367). Therefore, we synthesized the data via a fixed-effects model. The pooled OR revealed a significant reduction in the risk of HBVr for patients in high HBV DNA group compared with those in low HBV DNA group (OR = 0.30, 95% CI 0.15–0.58, Figure 4).

We performed subgroup analyses to investigate the influence of risk factors on HBVr. As for cut-off value, higher HBV DNA was associated with lower HBVr when cut-off value was 500 IU/mL (OR = 0.27, 95% CI 0.07–0.98) and 2000 IU/mL (OR = 0.13, 95% CI 0.04–0.49). With respect to antiviral treatment, in the subgroup where all patients received antiviral therapy, the high HBV DNA group exhibited a lower risk of HBVr than the low HBV DNA group did (OR = 0.42, 95% CI 0.18–0.98). When partial patients underwent antiviral treatment, there was no difference in the rate of HBVs between high HBV DNA and low HBV DNA (OR = 0.36 95% CI 0.04–2.89). The results of the subgroup analyses are shown in Table 4 and Supplementary Figure S5.

**Table 4:** Subgroup analyses of baseline HBV DNA level for HBVr.

Subgroup	No. of studies	Sample size	Effects model	OR and 95%CI	Heterogeneity	
					I^2^ (%)	*P* value
HBVr	8	1,151	Fixed	0.30 (0.15–0.58)	8.2	0.367
Treatment method						
Combination therapy	5	671	Fixed	0.15 (0.05–0.44)	0	0.464
Mixed	3	480	Fixed	0.60 (0.25–1.46)	0	0.378
Cut-off value						
500	2	351	Fixed	0.27 (0.07–0.98)	0	0.699
2,000	4	522	Fixed	0.13 (0.04–0.49)	17.8	0.302
Antiviral treatment						
All	4	639	Random	0.42 (0.18–0.98)	0	0.829
Partial	4	512	Random	0.36 (0.04–2.89)	50.7	0.107

### 3.7 Sensitivity analysis and publication bias

**Figure 5:**
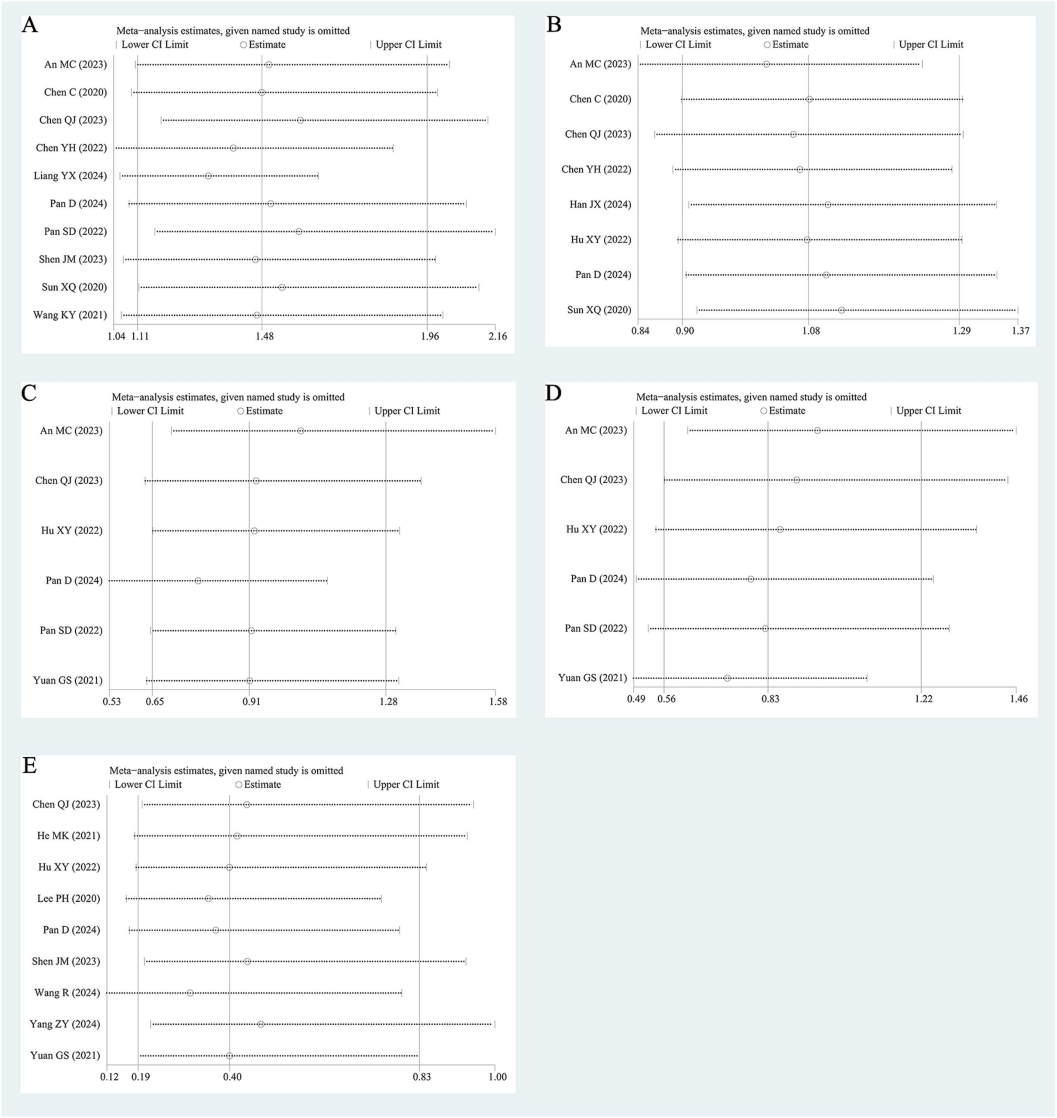
Sensitivity analysis of OS **(A)**, PFS **(B)**, ORR **(C)**, DCR **(D)**, HBVr **(E)**.

As shown in Figure 5, the combined HR and combined OR remained consistent with the aforementioned results after one single study was excluded at a time, suggesting that the results of the precent review were relatively stable.

The funnel plots for PFS, DCR, and HBVr displayed symmetry. However, the funnel plots for OS and ORR showed asymmetry (Supplementary Figure S6). Furthermore, there was no significant publication bias according to Egger’s test and or Begg’s test for OS (Egger’s test: *p* = 0.056, Begg’s test: *p* = 0.107), PFS (Egger’s test: *p* = 0.642, Begg’s test: *p* = 0.711), ORR (Egger’s test: *p* = 0.760, Begg’s test: *p* = 1.000), DCR (Egger’s test: *p* = 0.260, Begg’s test: *p* = 0.3000) or HBVr (Egger’s test: *p* = 0.710, Begg’s test: *p* = 1.000), as illustrated in Supplementary Figure S7.

## 4 Discussion

PD-1 and CTLA-4 suppress cytotoxic T cells and play critical roles in preventing the destruction of virus-infected hepatocytes (Cho et al., 2017). Thus, immunotherapy via interference with the PD-1/PD-L1 axis may cause hepatocyte destruction and previously latent viruses may be released into the circulation (Cho et al., 2016; Knolle and Thimme, 2014). These mechanisms disclosed that patients with high HBV DNA might undergo HBV reactivation and liver injury during ICIs therapy. For this reason, many clinical trials have excluded the patients with high HBV DNA (El-Khoueiry et al., 2017; Finn et al., 2020). However, numerous patients with high-level require ICIs to prolong survival in clinical practice. Therefore, it is crucial to clarify the safety and effectiveness of immunotherapy in liver cancer patients with high HBV DNA level. This meta-analysis included 17 studies with 2,130 patients to comprehensively compare the efficacy and safety of immunotherapy between high baseline HBV DNA group and low baseline HBV DNA group. No difference in PFS (HR = 1.08, 95% 0.90–1.29), ORR (OR = 0.91, 95% 0.65–1.28) or DCR (OR = 0.83, 95% 0.58–1.20) was observed in patients with high HBV DNA and low HBV DNA. However, patients with high HBV DNA had worse OS than those with low HBV DNA did (HR = 1.48, 95% CI: 1.11–1.96).

The human papilloma viral load has been reported to influence the clinical outcomes of ICIs therapy for virus-dependent anal squamous cell carcinoma (Balermpas et al., 2017). Data suggested that patients with high baseline HBV DNA level have unfavorable impact on prognosis of liver cancer after hepatic resection (Yang et al., 2012; Sohn et al., 2014; Sun et al., 2021). Zhou’s meta-analysis, which evaluated the impact of HBV DNA level on post-hepatectomy recurrence of HBV-related HCC, suggested that high viral load was associated with poorer OS (Zhou et al., 2014). In patients with advanced HBV-related HCC who were treated with sorafenib, a high initial HBV load was recognized as a detrimental prognostic factor for survival (Yang et al., 2015). Our results also revealed that high HBV DNA level was associated with poor OS in liver cancer patients treated with ICIs. The exact mechanism by which high HBV DNA lead to poor OS was unclear. One possible mechanism was that adhesion molecules on the sinusoidal cells were upregulated in patients with high HBV DNA, which in turn may enhance tumor progression and spread (Yang et al., 2012). Another reason could be that, unlike other tumors, the prognosis of liver cancer patients was associated not only with the status of intrahepatic tumors but also with liver function (Yu and Kim, 2014). A previous study reported that the significant impact of elevated HBV DNA on OS was linked to both tumor-related mortality and liver-related mortality. Patients with high HBV DNA were confirmed to have a higher liver-related mortality rate (Yang et al., 2012). Furthermore, it has been reported that tumor-infiltrating HBV-specific CD8+ T cells are related to improved survival outcomes (Cheng et al., 2021). The reduced OS observed in high levels of HBV DNA group might be associated with excessive depletion of specific CD4+ T cells and CD8+ T cells caused by high HBV DNA (Kalathil and Thanavala, 2021).

Notably, the result of subgroup analysis based on antiviral therapy revealed that patients with high HBV DNA and patients with low HBV DNA had similar OS when all patients were treated with antiviral therapy (HR = 1.51, 95% CI: 0.85–2.68), which indicated that antiviral therapy may have an effect on prognosis. Our results was consistent with the conclusion of another meta-analysis conducted by Ji et al. (2024). However, it is noted that the studies we included differed from those selected by Ji et al. Specifically, Ji’s meta-analysis was restricted to patients with HCC and did not include those with ICC. Furthermore, Ji’s inclusion criteria was limited to studies involving ICIs either as monotherapy or in combination with targeted drug, excluding those that incorporated chemotherapy or radiotherapy. Additionally, the sample size of the studies included by Ji et al. (2024) exceeded 40 cases, while our meta-analysis did not limit the sample size. In previous studies, antiviral therapy has been reported to be effective in improving prognosis of HCC patients. Yang et al. reported that antiviral therapy can improve the prognosis of patients with high HBV DNA in comparison with those who did not receive antiviral therapy in HBV-related HCC (Yang et al., 2012). The disparate effects of antiviral therapy on the prognosis of patients in the high and low groups were elucidated by Yang’s research. In the absence of antiviral treatment, patients with elevated baseline HBV DNA level continued to exhibit high viral loads and patients with elevated baseline HBV DNA level maintained a high viral load during therapy. Consequently, patients with high baseline HBV DNA level demonstrated significantly worse survival outcomes than those with low baseline HBV DNA levels due to the adverse effects of high viral loads on prognosis. With the administration of antiviral therapy, there was a significant enhancement in survival outcomes for high HBV DNA group and low HBV DNA group; nevertheless, this improvement was more pronounced in the high HBV DNA group than in the low HBV DNA group (Yang et al., 2015).

Although antiviral therapy had a more significant influence on patients with high HBV DNA level, it was worth noting that effective antiviral treatment was also of significance for patients with low HBV DNA patients. In Sun’s study, 7 patients from the low group demonstrated a significant increase in HBV DNA along with poorer prognosis. Among them, 4 patients did not receive antiviral treatment, indicating that low HBV DNA was a risk factor for the prognosis of HCC in the absence of effective antiviral treatment (Sun et al., 2021). Among patients with low HBV DNA levels, antiviral therapy markedly decreased HCC recurrence (Huang et al., 2018). Consequently, antiviral prophylaxis is recommended for patients with HBV-related HCC, regardless of their HBV DNA levels (Terrault et al., 2018).

In addition to antiviral treatment, the cut-off of HBV DNA was vital for assessing the influence of HBV DNA on the effectiveness of ICIs. The two subgroups based on cut-off value for OS showed different outcomes. At cut-off value of 2000 IU/mL, patients with high HBV DNA experienced poorer OS compared to those with low HBV DNA. In contrast, no significant difference was observed when the cut-off value was set at 500 IU/mL. The KEYNOTE-224 trial (Zhu et al., 2018) and the CheckMate-40 trial (El-Khoueiry et al., 2017) stipulated that HCC patients should have an HBV load of less than 100 IU/mL prior to receiving their first dose of ICI. However, in the IMbrave 150 trial (Finn et al., 2020), the threshold was set at 500 IU/mL. The cut-off values reported in the included studies exhibited variability. Our subgroup analyses focused on the frequently cited cut-off values of 500 IU/mL and 2000 IU/mL; however, because of insufficient data, we could not assess the effects of other cut-off values on the outcomes. Additional large-scale prospective studies are needed to determine the optimal cut-off value for HBV DNA.

Regarding HBVr, in our study, the group with high HBV DNA presented a lower rate of HBVr in comparison with the group with low HBV DNA. This may be attributed to the fact that HBV DNA detection was a standard procedure for HCC patients, and when serum HBV DNA levels exceeded the normal range, adequate antiviral treatment, which can markedly attenuate the risk of viral reactivation and augment the liver function reserve, is initiated to prevent HBV reactivation (Li et al., 2020). With respect to patients presenting with undetectable or baseline HBV DNA, a number of physicians asserted that antiviral prophylaxis can be safely omitted (Zhang et al., 2019). Subgroup analysis indicated that when all patients received antiviral treatment, those with high HBV DNA level still had a lower HBVr rate compared to the low HBV DNA level. These findings suggested that antiviral treatment significantly reduced the HBVr in the high HBV DNA group. High baseline HBV DNA should not be an absolute contraindication to ICIs in liver cancer patients receiving antiviral treatment.

There were also several factors that influenced heterogeneity. The timing of initiating antiviral therapy was diverse in the included studies. In some of the studies, patients had already initiated antiviral therapy prior to immunotherapy, whereas in other studies, patients initiated antiviral therapy during the period of ICIs. In addition, recent research indicated that patients treated with tenofovir had a lower risk of HCC occurrence and recurrence compared to those receiving entecavir (Choi et al., 2019; Choi et al., 2021); however, later studies reported no significant difference in HCC risk between the two groups (Kim et al., 2019; Lee, 2020). The types of antiviral therapy differed, which may lead to heterogeneity. Hence, the influence of antiviral therapy on outcomes ought to should be interpreted with caution. Moreover, the type of ICIs and their combination treatment strategies were complex among the included studies, which might influence the outcomes. Finally, given the type of liver cancer, HCC and ICC, two subtypes of primary liver cancer, differ in terms of cellular origins, morphology, metastatic capacity, treatment methods, prognosis, and immune microenvironments (Jiang et al., 2024). In our analysis, with the exception of one study that encompassed patients with HCC and ICC, all the remaining studies included patients with ICC, making it difficult to assess the impact of the type of liver cancer on the results.

Liu et al. (2018) study demonstrated that genotype B was prevalent in southern China, while Genotype C in northern China. Furthermore, the research highlighted that chronic hepatitis B patients born in regions between southern and northern China had a higher likelihood of carrying B/C intergenotypic recombinants. Notably, compared to the parental genotypes B or C, the B/C intergenotypic recombinants exhibited significantly higher levels of viral DNA load. Different genotypes may lead to varying DNA viral load, and none of the studies included in our meta-analysis investigated the impact of genotype on the level of HBV DNA, which constituted a significant limitation of our study.

Potential limitations of the present meta-analysis included the following. First, all included studies were retrospective cohort studies. Therefore, the potential for selection of the patients cannot be overlooked. Second, the timing of HBV DNA screening varied among the enrolled patients because of the retrospective nature of the studies, which could introduce bias in the detection rate of HBVr. Third, all the included studies were conducted in China, which was mainly attributed to the high incidence of HBV-HCC in China, and the applicability of the results to other populations was yet to be determined. Finally, in this meta-analysis, all patients, except those who received anti-PD-L1 blockade, were treated with anti-PD-1 blockade. Additional studies are needed to assess the relevance of our findings to other ICIs.

## 5 Conclusion

In conclusion, for liver cancer patients treated with ICIs, high HBV DNA was associated with worse OS, not with PFS, ORR, or DCR. However, subgroup analysis revealed that baseline HBV DNA level had no impact on the prognosis of liver cancer patients receiving ICIs in combination with antiviral therapy. Furthermore, the risk of HBVr in the high HBV-DNA group was lower than that in the low HBV DNA group, particularly in patients who received antiviral therapy. HBVr should not be a contradiction for ICIs therapy among patients under the protection of antiviral therapy. Considering the limitations of our meta-analysis, the results require further verification through prospective studies with larger sample sizes.
