# Supplementary material for: Association of hepatitis B virus DNA levels with efficacy and safety and the impact of antiviral therapy on prognosis in liver cancer patients receiving immune checkpoint inhibitors therapy: a systematic review and meta-analysis
Source: Front Microbiol. 2025 Jan 22;16:1501139. doi: 10.3389/fmicb.2025.1501139 (PMC11794511; doi:10.3389/fmicb.2025.1501139)
Supplement: Supplementary file 1 [file Table_1.doc]

**Supplementary Table 1.** Search strategy

| **PubMed** | **Searching Strategy** | **Results** |
| --- | --- | --- |
| #1 | "Neoplasms"[MeSH Terms] OR "Tumor"[Title/Abstract] OR "Neoplasm"[Title/Abstract] OR "Tumors"[Title/Abstract] OR "Neoplasia"[Title/Abstract] OR "Neoplasias"[Title/Abstract] OR "Cancer"[Title/Abstract] OR "Cancers"[Title/Abstract] OR "malignant neoplasm"[Title/Abstract] OR "Malignancy"[Title/Abstract] OR "Malignancies"[Title/Abstract] OR "malignant neoplasms"[Title/Abstract] OR "neoplasm malignant"[Title/Abstract] OR "neoplasms malignant"[Title/Abstract] OR "benign neoplasms"[Title/Abstract] OR "benign neoplasm"[Title/Abstract] OR "neoplasms benign"[Title/Abstract] OR "neoplasm benign"[Title/Abstract] | 5153810 |
| #2 | "Immune Checkpoint Inhibitors"[MeSH Terms] OR "Immune Checkpoint Inhibitor"[Title/Abstract] OR "Immune Checkpoint Blocker"[Title/Abstract] OR "Immune Checkpoint Blockade"[Title/Abstract] OR "Immune Checkpoint Inhibition"[Title/Abstract] OR "pd l1 inhibitor*"[Title/Abstract] OR "Programmed Death Ligand 1 Inhibitor"[Title/Abstract] OR "PD-1-PD-L1 Blockade"[Title/Abstract] OR "CTLA-4 Inhibitor"[Title/Abstract] OR "Cytotoxic T Lymphocyte Associated Protein 4 Inhibitor"[Title/Abstract] OR "PD-1 Inhibitor"[Title/Abstract] OR "Programmed Cell Death Protein 1 Inhibitor"[Title/Abstract] OR "nivolumab"[Title/Abstract] OR "pembrolizumab"[Title/Abstract] OR "atezolizumab"[Title/Abstract] OR "camrelizumab"[Title/Abstract] OR "cemiplimab"[Title/Abstract] OR "durvalumab"[Title/Abstract] OR "toripalimab"[Title/Abstract] OR "Tislelizumab"[Title/Abstract] OR "Penpulimab"[Title/Abstract] OR "Sintilimab"[Title/Abstract] OR "zimberelimab"[Title/Abstract] OR "Sugemalimab"[Title/Abstract] OR "PD-1"[Title/Abstract] OR "PD-L1"[Title/Abstract] OR "Ipilimumab"[Title/Abstract] OR "Opdivo"[Title/Abstract] OR "keytruda"[Title/Abstract] OR "Imfinzi"[Title/Abstract] OR "avelumab"[Title/Abstract] OR "Bavencio"[Title/Abstract] OR "cemiplimab"[Title/Abstract] OR "SHR-1210"[Title/Abstract] OR "Tislelizumab"[Title/Abstract] OR "dostarlimab"[Title/Abstract] OR "envafolimab"[Title/Abstract] OR "KN035"[Title/Abstract] OR "Sugemalimab"[Title/Abstract] | 76039 |
| #3 | " Hepatitis B virus "[MeSH Terms] OR "Hepatitis B Virus"[Title/Abstract] OR "HBV"[Title/Abstract] OR "Chronic Hepatitis B Virus Infection"[Title/Abstract] OR "Chronic Hepatitis B"[Title/Abstract] OR "hepatitis b virus infection chronic"[Title/Abstract] OR "resolved hepatitis B"[Title/Abstract] OR "hepatitis B surface antigen"[Title/Abstract] OR "hepatitis B core antigen"[Title/Abstract] OR "hepatitis B core antibody"[Title/Abstract] OR "HBsAg"[Title/Abstract] OR "HBcAb"[Title/Abstract] OR "HBeAg"[Title/Abstract] OR "HBV DNA"[Title/Abstract] OR "Hepatitis B Virus DNA"[Title/Abstract] | 83571 |
| #4 | #1 AND #2 AND #3 | 311 |

**Supplementary Table 2.** Quality assessment of cohort studies by Newcastle–Ottawa Scale (NOS)

| Author | Selection | Comparability | Outcome | Toal |
| --- | --- | --- | --- | --- |
| An MC | 3 | 2 | 2 | 7 |
| Chen C | 3 | 2 | 3 | 8 |
| Chen QJ | 3 | 2 | 3 | 8 |
| Chen YH | 3 | 0 | 2 | 5 |
| Han JX | 3 | 0 | 2 | 5 |
| He MK | 3 | 2 | 2 | 7 |
| Hu XY | 3 | 0 | 2 | 5 |
| Lee PH | 3 | 0 | 3 | 6 |
| Liang YX | 3 | 0 | 3 | 6 |
| Pan D | 3 | 0 | 2 | 5 |
| Pan SD | 3 | 0 | 2 | 5 |
| Shen JM | 3 | 0 | 2 | 5 |
| Sun XQ | 3 | 0 | 3 | 6 |
| Wang KY | 3 | 2 | 2 | 7 |
| Wang R | 3 | 2 | 3 | 8 |
| Yang ZY | 3 | 2 | 2 | 7 |
| Yuan GS | 3 | 2 | 2 | 7 |


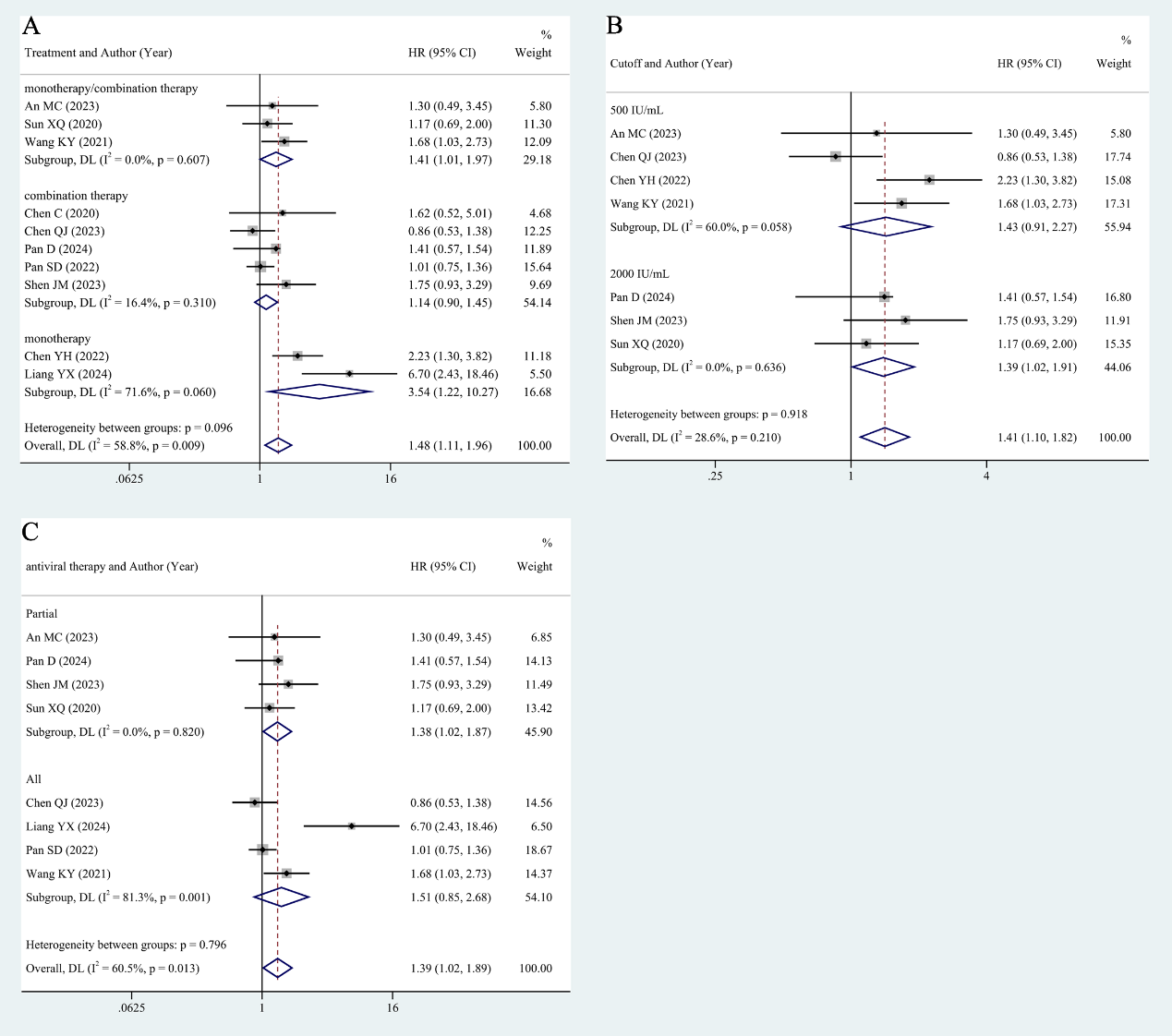


**Supplementary Figure. 1.** Subgroup analysis of OS, stratified by: (A) treatment method; (B) Cut-off value; (C) Antiviral treatment


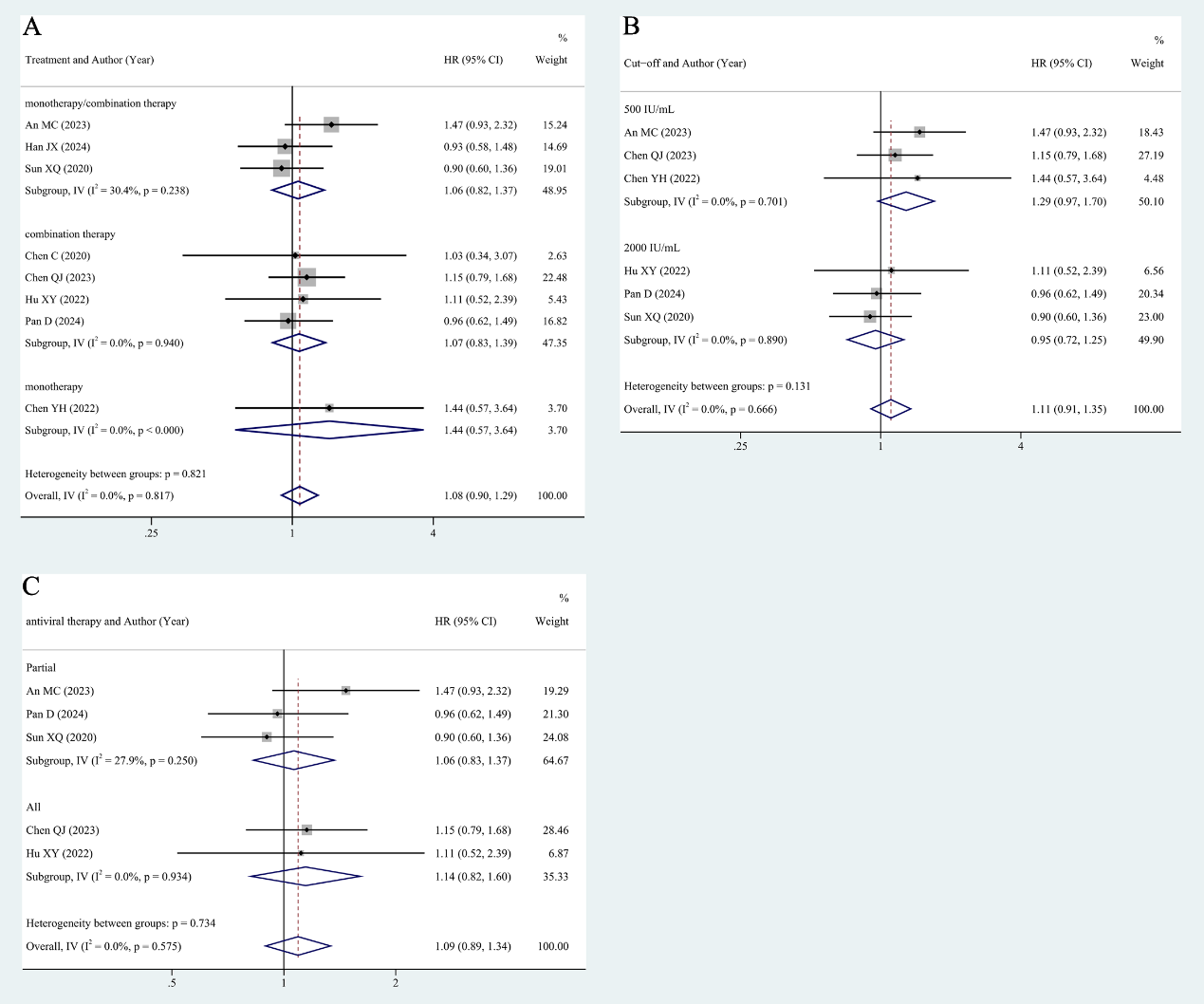


**Supplementary Figure. 2.** Subgroup analysis of PFS, stratified by: (A) treatment method; (B) Cut-off value; (C) Antiviral treatment


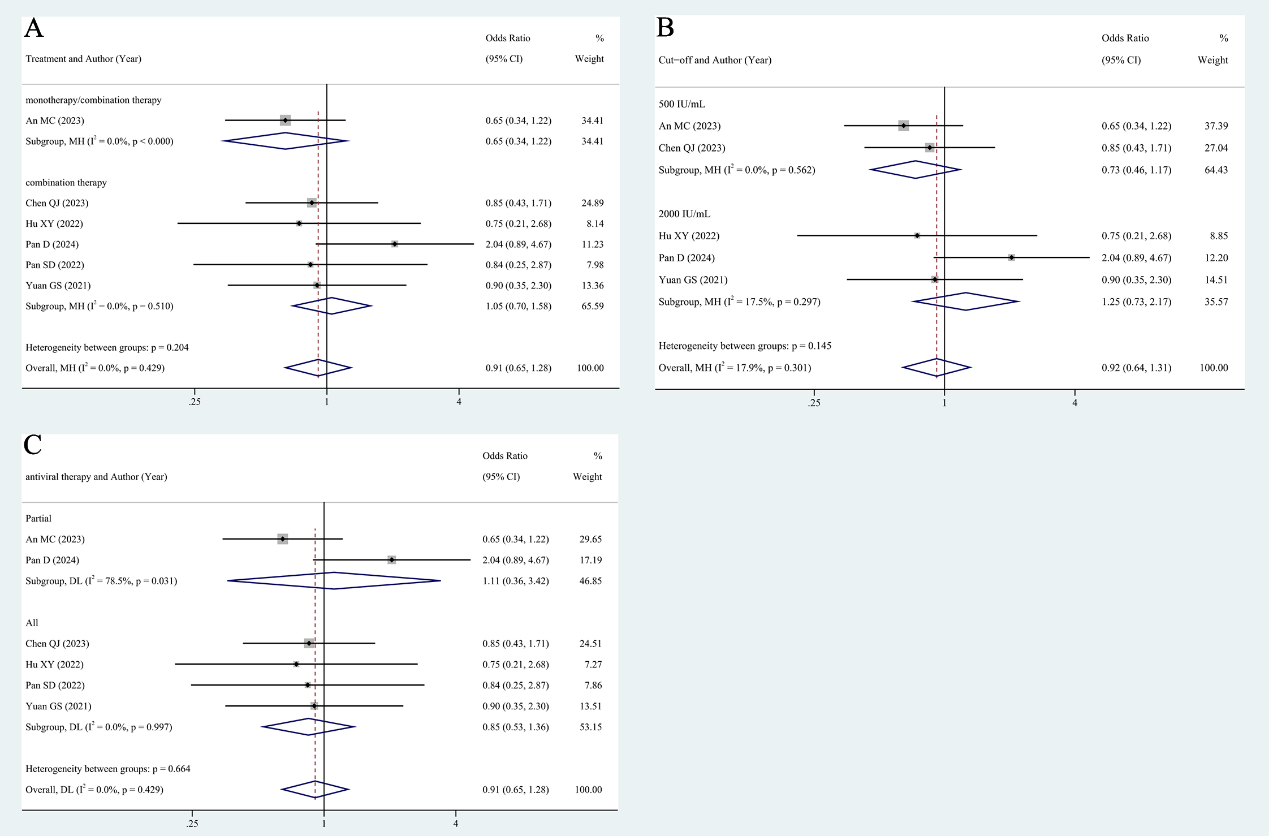


**Supplementary Figure. 3.** Subgroup analysis of ORR, stratified by: (A) treatment method; (B) cut-off value; (C) antiviral treatment


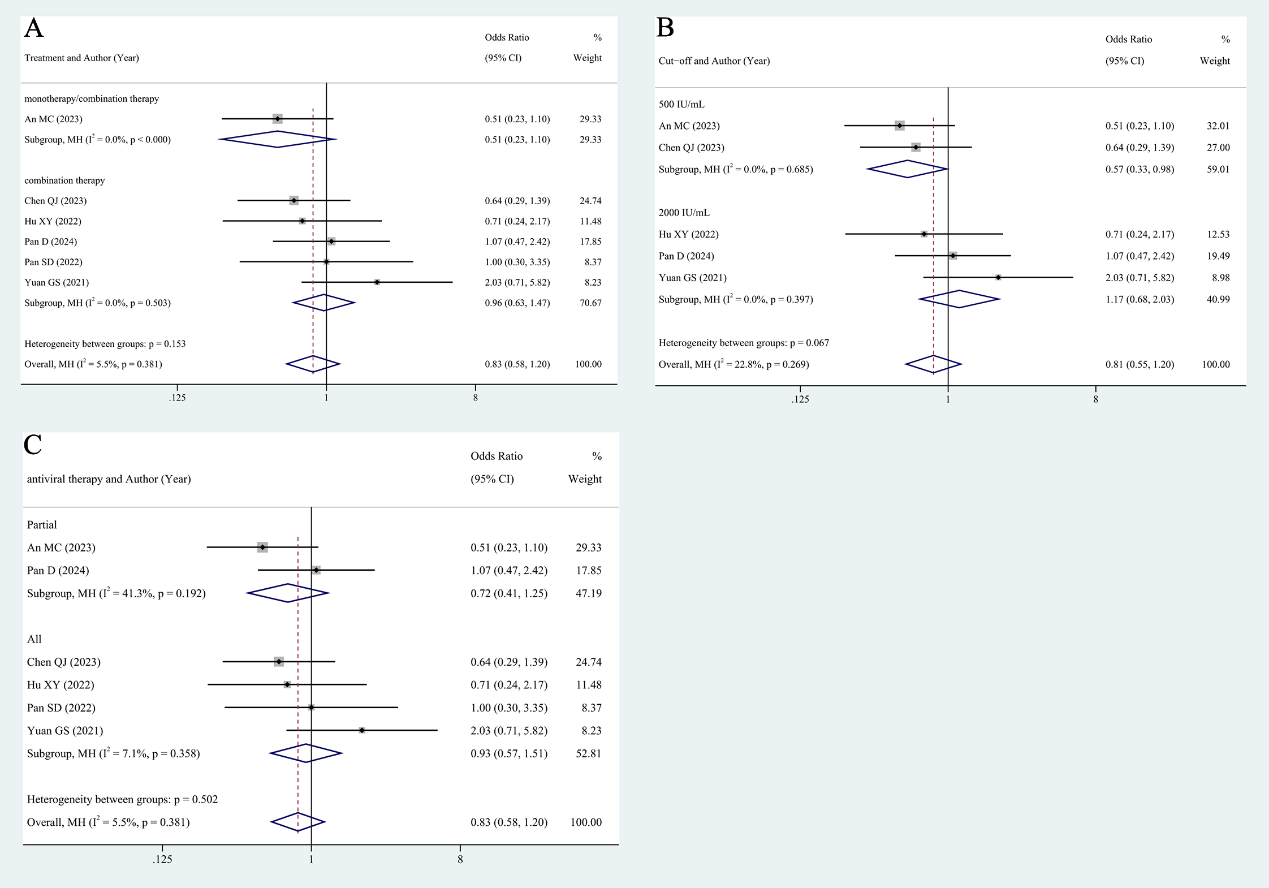


**Supplementary Figure. 4.** Subgroup analysis of DCR, stratified by: (A) treatment method; (B) cut-off value; (C) antiviral treatment

**
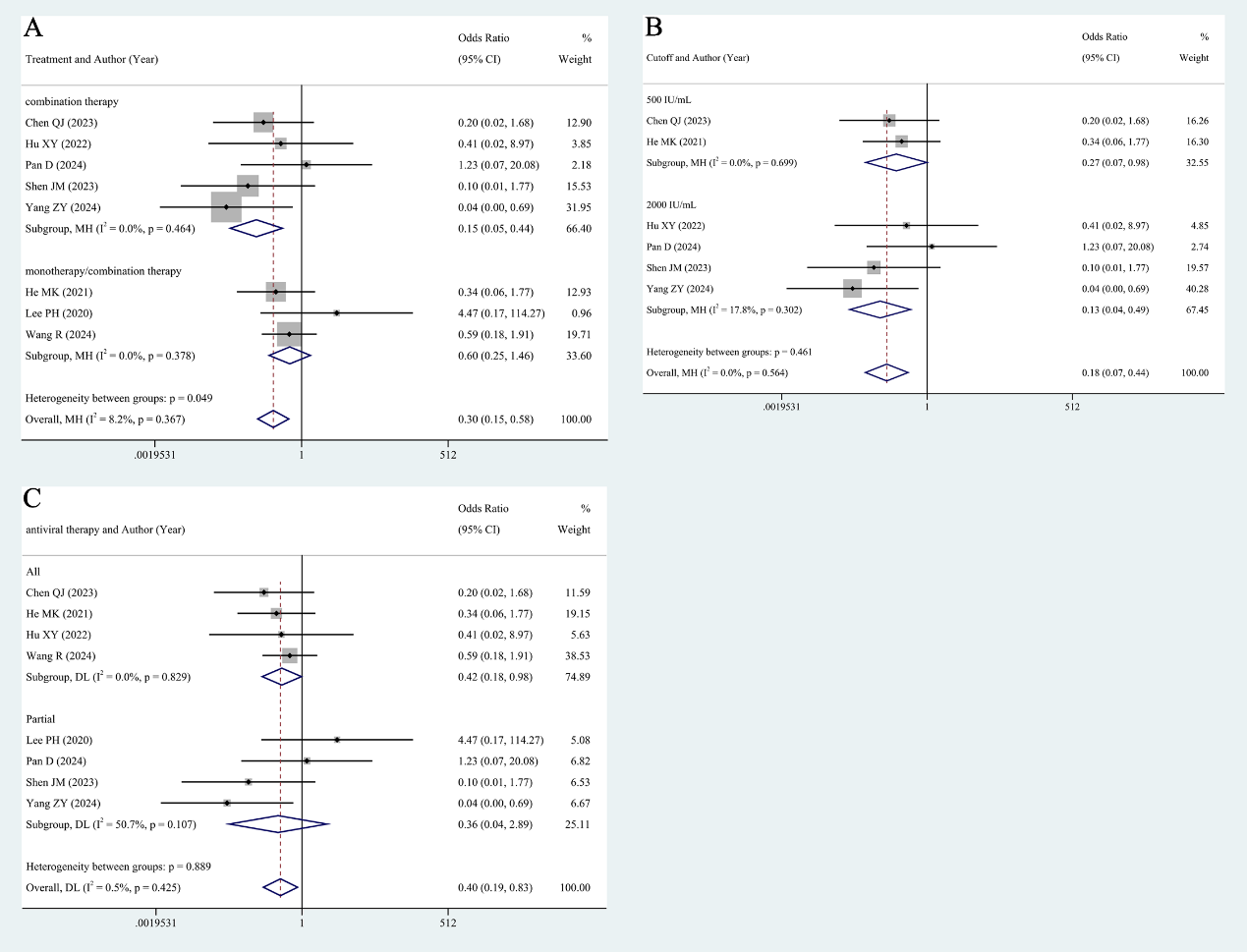
**

**Supplementary Figure. 5.** Subgroup analysis of HBVr, stratified by: (A) treatment method; (B) cut-off value; (C) antiviral treatment

**
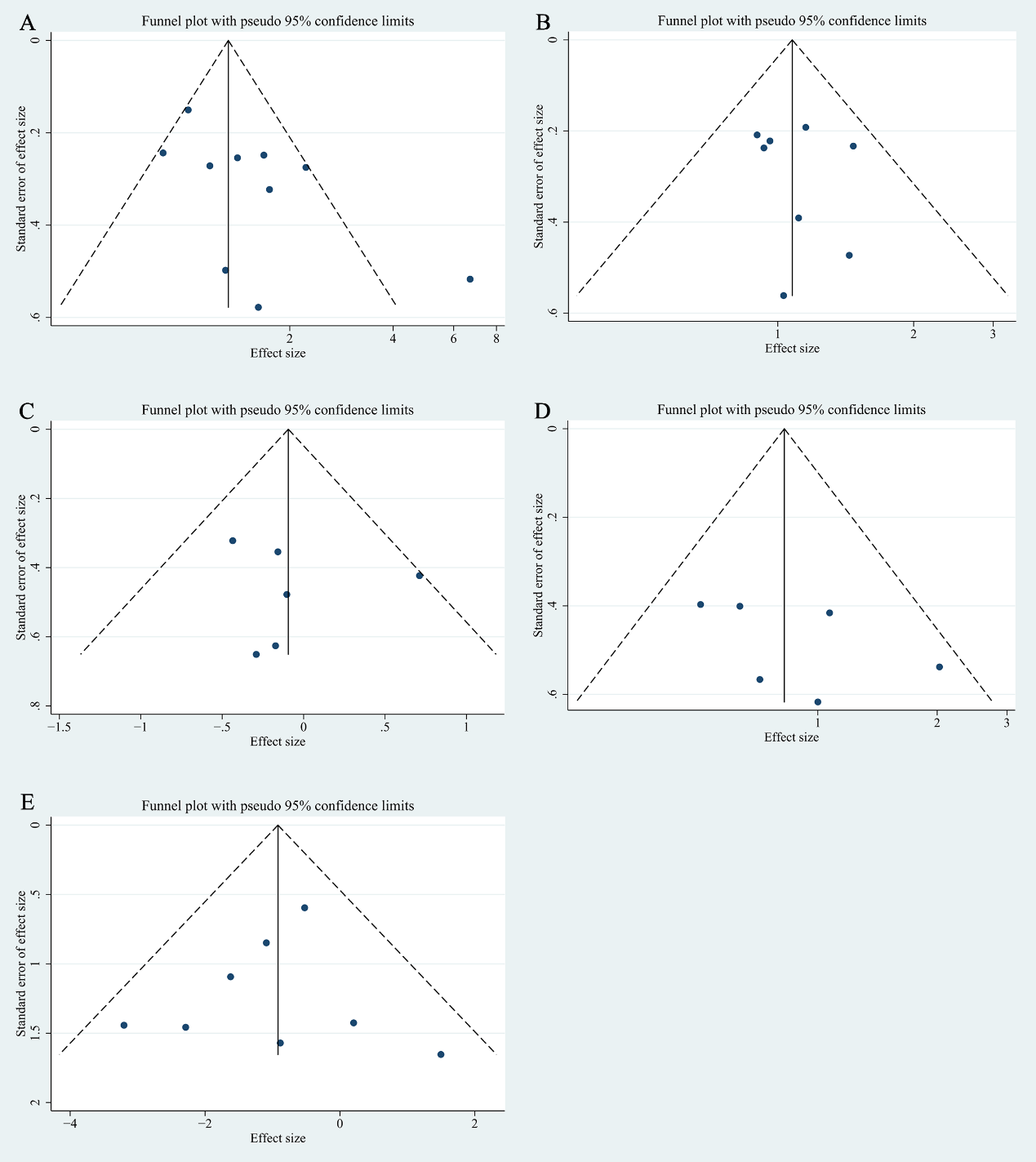
**

**Supplementary Figure. 6.** Funnel plots of OS (A), PFS (B), ORR (C), DCR (D), HBVr (E)

**
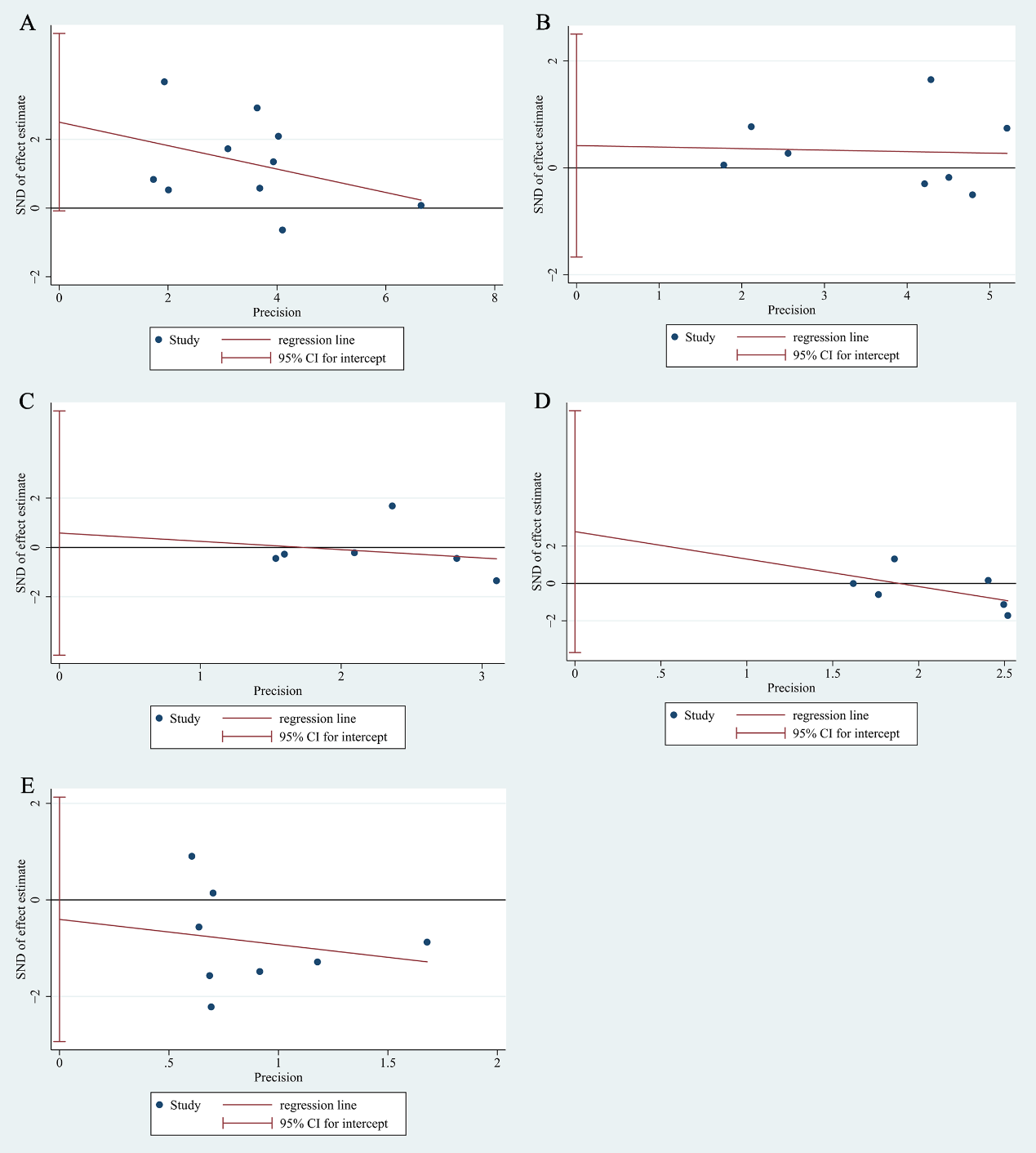
**

**Supplementary Figure. 7.** Egger’s test of OS (A), PFS (B), ORR (C), DCR (D), HBVr (E)
